# Effects of the Combined Supplementation of Caffeine and Rhodiola Rosea with Resistance Training on Lower Limb Explosive Power in Male Volleyball Players

**DOI:** 10.3390/nu17040681

**Published:** 2025-02-14

**Authors:** Zhaolong Wang, Haoyu Du, Huixin Li, Kai Zhao, Bingran Zhao, Yifei Ma, Jiashuo Zhang, Kangshuo Wu, Wei Jiang, Chang Liu

**Affiliations:** 1 Chinese College of Volleyball, Beijing Sport University, Beijing 100084, China; wangzhaolong@bsu.edu.cn (Z.W.); foresight195@gmail.com (H.D.); 2326@bsu.edu.cn (K.Z.); 15101004388@163.com (B.Z.); 13226410865@163.com (Y.M.); 18504110517@163.com (J.Z.); 13478588952@163.com (K.W.); 2 School of Sport Science, Beijing Sport University, Beijing 100084, China; 2021011319@bsu.edu.cn

**Keywords:** Rhodiola rosea, caffeine, lower limb explosive power, fatigue recovery, volleyball players

## Abstract

Background: This study investigated the effects of combined Rhodiola rosea (RHO) and caffeine (CAF) supplementation on lower limb explosive power and fatigue recovery in volleyball players, aiming to validate their potential synergistic effects. Methods: A randomized, double-blind, placebo-controlled design was implemented involving 48 male volleyball athletes allocated into four groups: placebo (CTR), Rhodiola rosea (RHO), caffeine (CAF), and combined (RHO + CAF). Over four weeks, participants engaged in twice-weekly high-intensity lower limb resistance training, with performance evaluated through multiple tests, including two-step-approach jump height, countermovement jump (CMJ), five-jump test (5 JT), continuous 20 vertical jumps, and intermittent jump recovery alongside Rate of Perceived Exertion (RPE) assessments. Results: Results showed that the combined supplementation (RHO + CAF) group exhibited significant improvement across multiple performance metrics. In particular, the combined group demonstrated the highest increases in jump height and the least power decline during high-frequency jumps, outperforming the CTR and other supplementation groups (*p* < 0.05). Additionally, RHO + CAF supplementation significantly lowered RPE scores, particularly in the final week of training, indicating improved perceived fatigue and recovery. Conclusions: This study suggests that combining RHO and CAF optimizes high-intensity performance by enhancing explosive power, reducing fatigue, and improving recovery, presenting an effective nutritional strategy for volleyball athletes.

## 1. Introduction

With the increasing pursuit of health and quality of life, the scientific community has been deeply exploring strategies to enhance sports performance and physiological adaptation. In this context, the potential of natural supplements in improving athletic performance has gained widespread attention. Rhodiola rosea (RHO) and caffeine (CAF) have garnered significant interest for their roles in enhancing muscular endurance and explosive power [1].

RHO is a recognized adaptogen valued for its fitness-enhancing properties. Its main active compound, salidroside, improves oxygen utilization, boosts antioxidant capacity, reduces fatigue, and accelerates recovery [2]. Studies indicate that RHO supplementation can reduce exercise-related pain and muscle damage, improve muscle recovery, enhance antioxidant defenses, decrease oxidative stress, lower RPE, and increase power without adding fatigue [2,3]. This makes RHO particularly beneficial for sports demanding sustained high-intensity output.

Caffeine is a natural nutrient found in coffee and tea and has been used for a long time to improve athletic performance [4]. Since the World Anti-Doping Agency (WADA) removed CAF from the list of prohibited substances in 2004, it has become a commonly used legal supplement among athletes [5]. CAF enhances performance by antagonizing adenosine receptors, which promotes the release of neurotransmitters such as dopamine and catecholamines (epinephrine and norepinephrine) [6]. Studies have confirmed that moderate CAF intake can enhance both anaerobic and aerobic exercise capacities, including muscle endurance, speed, and explosive power [7].

Although there have been numerous individual studies on the potential effects of RHO and CAF, limited understanding exists on whether their combined application produces greater effects. It has been reported that the AMPK/Sirt1/PGC-1α pathway is a key regulatory factor for mitochondrial biogenesis [8,9,10,11]. Salidroside regulates mitochondrial biogenesis via the Sirt1/PGC-1α axis, and PGC-1α, as a downstream component of the AMPK pathway, plays a central role in regulating mitochondrial biogenesis, possibly affecting energy metabolism and muscle endurance [12,13,14]. This suggests that RHO may positively influence energy metabolism and muscle endurance through this pathway. On the other hand, CAF enhances performance by increasing central nervous system arousal, which is achieved by antagonizing adenosine receptors, thereby promoting neurotransmitter release [6]. Previous studies suggest that even high-intensity resistance training performed two to three times per week can significantly upregulate mitochondrial biogenesis markers, such as PGC-1α. This training regimen effectively activates key signaling pathways, including PGC-1α, thereby promoting mitochondrial biogenesis [9,15,16]. Given these mechanisms, it is essential to explore whether the combination of RHO and CAF, in conjunction with resistance training, can provide synergistic benefits in athletic performance.

Volleyball is a high-intensity sport event requiring athletes to frequently jump, react quickly, and produce repeated power outputs, particularly in key technical actions such as spiking and blocking [17]. These demands place a significant emphasis on lower limb explosive power and fatigue recovery ability. However, existing research mainly focuses on endurance sports or short-term strength training, with limited attention given to sports like volleyball that require a combination of power and endurance. Furthermore, while CAF’s performance-enhancing effects are well recognized, research on the effects of RHO on lower limb explosive power and recovery in volleyball players is still scarce.

The main objective of this study is to explore the potential synergistic effects of RHO and CAF on lower limb explosive power and fatigue recovery in volleyball players. In addition to supplementation, all participants underwent a lower limb resistance training regimen to assess the combined effects of supplementation and resistance training on performance. By adopting a comprehensive approach, including age-matched human participants, we aim to determine whether combined application yields better results than individual supplementation. Exploring these synergistic effects is significant not only for athletes but also provides a safe and effective strategy to optimize exercise performance and physiological adaptation for a wider population.

Primary hypothesis: Combined supplementation of RHO and CAF, along with resistance training, will significantly improve lower limb explosive power in volleyball players compared to placebo, RHO alone, or CAF alone.

Secondary hypothesis: Combined supplementation will accelerate fatigue recovery, allowing athletes to maintain a higher level of performance during high-intensity intermittent exercise, especially in volleyball matches.

## 2. Materials and Methods

### 2.1. Participants

A total of 48 male volleyball athletes (Chinese first-class athletes) aged 18 to 23 years (Table 1), each with at least two years of professional training experience, were recruited for this study. Prior to the experiment, researchers provided a detailed explanation of the study objectives, procedures, potential benefits, and risks, obtaining written informed consent from all participants. Additionally, participants completed the Physical Activity Readiness Questionnaire (PAR-Q), with all results indicating that they were cleared for physical activity with no significant health concerns identified. Participants were also instructed to maintain dietary records to ensure the effective monitoring of their diet and health status throughout the study period.

To ensure participant health and safety, a comprehensive health assessment was conducted, confirming that none of the participants had a history of major illnesses or allergies to RHO or CAF. Participants were instructed to abstain from caffeine-containing foods and beverages and to avoid strenuous exercise for 72 h prior to data collection to minimize external influences.

Participants were randomly assigned to one of the four experimental groups (*n* = 12 per group) using a randomization process in Microsoft Excel. Random numbers were generated for each participant using the RANDBETWEEN (1, 4) function, which allocated them to one of the four groups. The allocation sequence was concealed to ensure a double-blind design, with both participants and researchers blinded to the group assignments.

### 2.2. Ethics Approval

The study was approved by the Ethics Committee of Beijing Sport University (approval number: 2024451H), adhering to the ethical principles of the Helsinki Declaration.

### 2.3. Study Design

**Figure 1:**
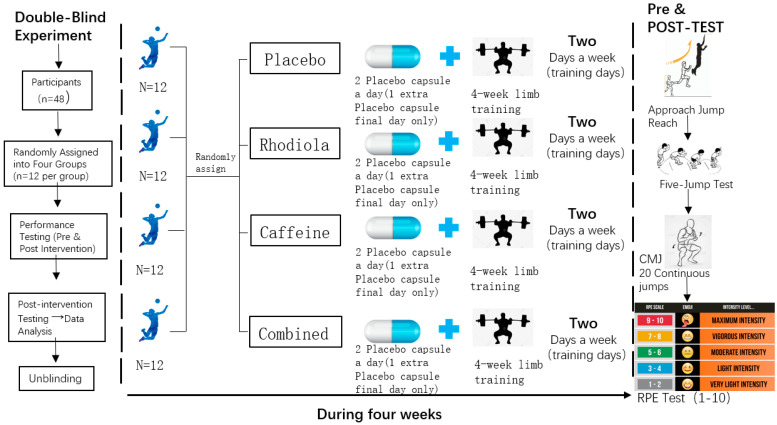
Experimental flowchart.

This study employed a four-week, randomized, double-blind, placebo-controlled design to evaluate the effects of RHO and CAF supplementation on lower limb explosive power and fatigue recovery in volleyball players, with all groups undergoing lower limb resistance training interventions (Figure 1). Testing was conducted at two time points: pre-test and post-test, following identical procedures.

Participants were randomly assigned to one of four groups (*n* = 12 per group) to ensure balanced group assignment. A double-blind procedure was applied, with both participants and researchers blinded to the supplementation assignments.

#### Supplementation Protocol

**Figure 2:**
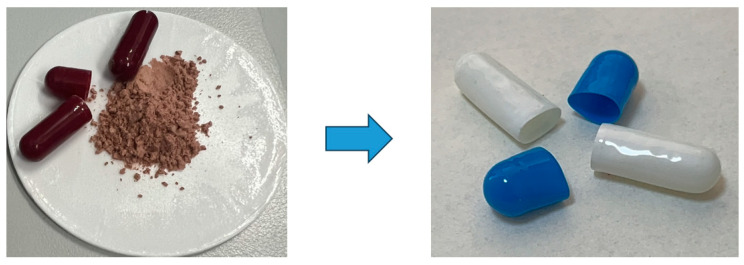
Preparation of placebo.

- Placebo Group (CTR): Consumed placebo capsules twice daily, identical in appearance to those of the RHO group to control for placebo effects. On the final test day, participants were administered a blank CAF capsule 30 min before testing to maintain consistency across all groups (Figure 2).
- Rhodiola rosea Group (RHO): Consumed RHO extract (2.4 g per day) in two divided doses, administered 30 min before breakfast and dinner. On the final test day, participants were administered a blank CAF capsule 30 min before testing to maintain consistency across all groups.
- Caffeine Group (CAF): Consumed CAF supplementation (3 mg per kg of body weight) 30 min before the final test day only. During the rest of the intervention period, participants consumed empty RHO capsules to maintain the double-blind design.
- Combined Group (RHO + CAF): Consumed RHO extract (2.4 g per day) in two divided doses, administered 30 min before breakfast and dinner throughout the intervention period, along with CAF supplementation (3 mg per kg of body weight) 30 min before the final test day only.

All supplements were distributed by the research team, ensuring identical capsule appearance to maintain blinding. Dosages were selected based on prior studies and safety guidelines [1].

### 2.4. Supplementation Preparation and Management

CAF supplements were provided in anhydrous CAF capsule form (200 mg per capsule, Nutricost, Vineyard, UT, USA). RHO supplements were supplied by Beijing Tongrentang Pharmaceuticals, China. All capsules contained identical excipients (rice flour, gelatin) to ensure uniform appearance across groups, maintaining blinding. During the experiment, participants were required to maintain stable dietary habits, avoiding additional supplements, especially caffeine-containing foods or beverages.

### 2.5. Training Intervention Program

To evaluate the effects of RHO and CAF supplementation on the lower limb strength and power of volleyball players, a four-week high-intensity lower limb resistance training program was designed. The training regimen referenced core exercises from established physical training guidelines, including the “NSCA-CSCS Essentials of Strength and Conditioning” and the “ACSM Guidelines for Exercise Testing and Prescription” textbooks.

The primary training movements included back squats, deadlifts, hip thrusts, and lunges for strength, alongside plyometric exercises like box jumps, depth jumps, and squat jumps to enhance explosive power (Table 2). Training was conducted twice weekly under the guidance of professional coaches, with loads and physiological indicators monitored to ensure safety.

### 2.6. Daily Dietary Intake Recording

Throughout the study period, all participants, regardless of training group, were instructed to maintain their regular diets. To further regulate habitual dietary intake, compliant food packaging options were provided, adhering to national food safety regulations. Dietary intake during the experiment was recorded in kilocalories (kcal), and daily intake of carbohydrates, proteins, and fats was quantified in grams (g). Notably, neither CAF nor RHO affected participants’ appetite or food intake. Participants were advised to continue their routine physical activities. Routine activities were monitored via a WeChat group, where daily step counts were recorded to document regular activity levels. Daily energy intake calculations followed the National Nutrient Database standards from the USDA to ensure consistency (https://fdc.nal.usda.gov/, accessed on 25 January 2025) [16].

### 2.7. Two-Step Approach Jump Height Test

**Figure 3:**
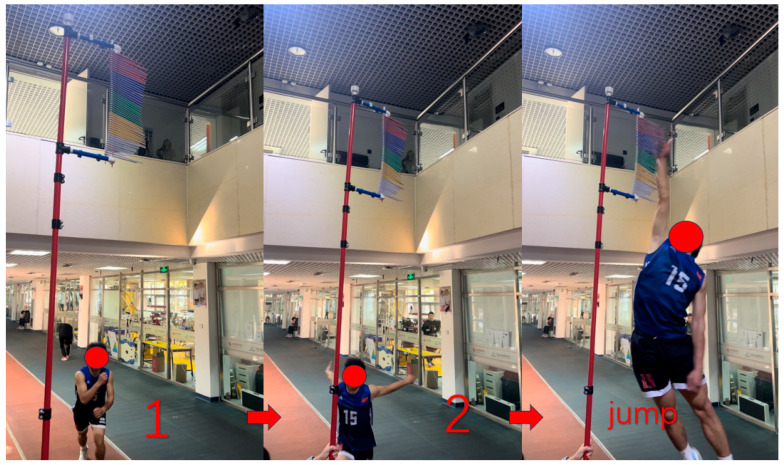
Two-step-approach jump height test.

The two-step-approach jump height test evaluates an athlete’s lower limb explosive power in situations similar to volleyball gameplay. In this test, participants start from a designated ready position, take a rapid two-step approach, and immediately jump to reach maximum height. Jump height is recorded using the Vertec Jump Measurement Device (Figure 3). Each participant completes three attempts with a one-minute recovery period between jumps, and the highest recorded jump height is used for data analysis.

### 2.8. Countermovement Jump (CMJ) Test

**Figure 4:**
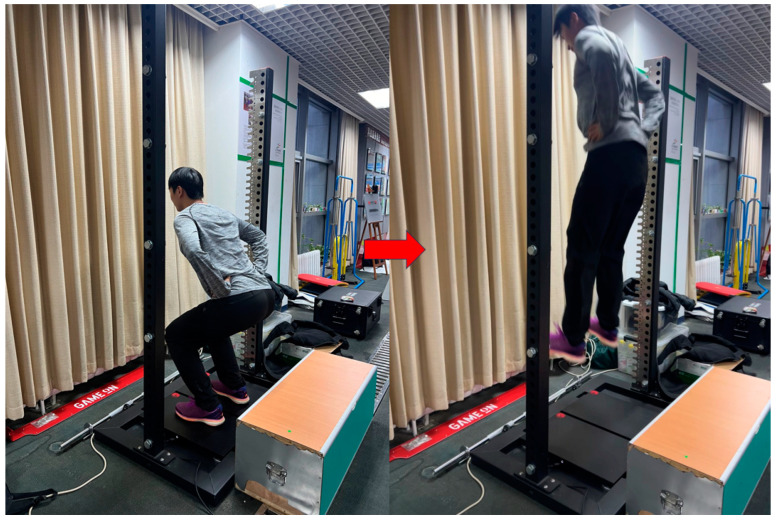
Countermovement jump test.

The CMJ test is widely used to assess explosive lower limb power and lower body strength in a vertical jump (Figure 4). Participants begin by standing with feet shoulder-width apart, looking forward with hands on hips, and positioning themselves at the center of a force plate. From this position, they perform a rapid downward movement by flexing the knees and hips, then immediately jump upward with maximum force. Throughout the jump, hand position remains unchanged, and the upper body stays upright. After each jump, a one-minute recovery period is observed. Three valid trials are recorded, and the maximum jump height (in centimeters) is used for analysis.

### 2.9. Five-Jump Test (5 JT)

**Figure 5:**
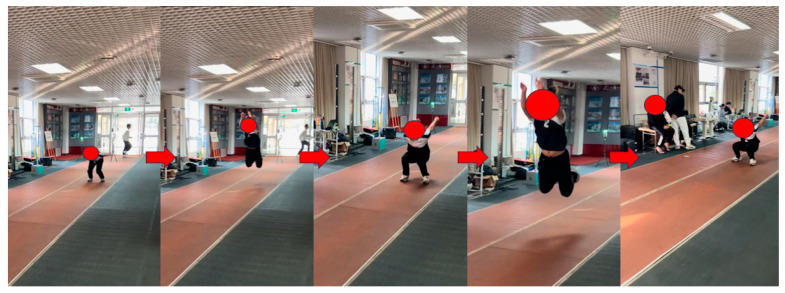
Five-jump test (5 JT).

The Five-Jump Test (5 JT) is used to assess lower limb muscle power through a series of horizontal jumps. Participants stand with feet shoulder-width apart at the starting line and perform five consecutive forward jumps, landing with both feet simultaneously after each jump. The total distance jumped across all five jumps is measured from the starting line to the closest point of contact on the landing. Each participant performs three attempts with the best distance recorded as the final score. This test measures both power and coordination in lower limb muscle groups (Figure 5).

### 2.10. Continuous 20 Vertical Jumps Test

The continuous 20 vertical jumps test measures sustained explosive power output under repeated jumping conditions. Participants begin by standing with their feet shoulder-width apart and their hands on their hips, maintaining an upright posture. They then perform a rapid downward movement by flexing their knees and hips, followed by a quick upward jump, reaching for maximum height each time. They are instructed to maintain a consistent rhythm and jump as high as possible each time. The average height across the 20 jumps is calculated using a Kistler force platform. This average height is used as the primary measurement to assess lower limb power endurance in repeated high-intensity efforts.

### 2.11. Intermittent Jump Recovery Test

The intermittent jump recovery test assesses fatigue recovery ability during repeated rounds of high-intensity jumping. Participants perform three rounds of 20 consecutive vertical jumps, with a mandatory 1.5 min rest period between each round. The procedure for each jump is the same as in the continuous 20 vertical jumps test. Each jump’s height is recorded, and the average jump height for each round is calculated. The total decline in average height across the rounds serves as an indicator of fatigue accumulation and recovery capability, providing insight into the effectiveness of supplementation on recovery.

### 2.12. Rate of Perceived Exertion (RPE) Scale

Participants rated their perceived exertion on a 1 to 10 scale (1 = no exertion, 10 = extremely strenuous) immediately after each test. RPE scores were used to assess the subjective effect of supplementation on fatigue perception and recovery. In this study, RPE was measured using two indicators: first, after completing three consecutive sets of 20 vertical jumps, RPE scores were recorded immediately, referred to as RPE (Round 3). The second indicator involved resistance training, where RPE scores were recorded after the first resistance training session and again after the second session of the fourth week, during a four-week period of bi-weekly resistance training and was designated as Week 4 Resistance Training RPE.

### 2.13. Baseline Data Collection

Baseline data were collected from all 48 participants prior to the intervention to assess the initial performance levels of the four groups (CTR, CAF, RHO, and CAF + RHO) across key metrics. These baseline measurements included the two-step-approach jump height, countermovement jump, five-jump, continuous 20 vertical jumps (20 J average (Avg) and 20 J 3rd round average), intermittent jump recovery, and Rate of Perceived Exertion scale for both the first week of resistance training and the 20 vertical jumps. Statistical analysis using one-way analysis of variance (ANOVA) revealed no significant differences between the groups for any of the baseline measurements (*p* > 0.05).

Specifically, the ANOVA results for the two-step-approach jump height showed no significant group differences (F(3,44) = 0.15, *p* = 0.9281). Similarly, no significant differences were found across the groups for the CMJ, 5 JT, and 20 J Avg (CMJ: F(3,44) = 0.27, *p* = 0.846; 5 JT: F(3,44) = 0.23, *p* = 0.876; 20 J Avg: F(3,44) = 1.22, *p* = 0.310). The 20 J 3rd round average test (20 J 3rd Round Avg), which assesses endurance and lower limb performance, also showed no significant differences (F(3,44) = 0.13, *p* = 0.942). Furthermore, the RPE scores for both the 20 J and the first week of resistance training revealed no significant group differences (20 J 3rd Round RPE: F(3,44) = 0.18, *p* = 0.913; 1st Week Resistance Training RPE: F(3,44) = 0.14, *p* = 0.937).

These findings indicate that all groups exhibited similar performance levels and perceived exertion prior to the intervention, confirming that the baseline data were comparable across groups and providing a solid foundation for evaluating the effects of the intervention.

### 2.14. Statistical Analysis

All data are presented as mean ± standard deviation (SD). Baseline values for all performance metrics are provided in Table 3. Group comparisons were performed using unpaired two-tailed *t*-tests for pairwise comparisons and one-way analysis of variance (ANOVA) with Dunnett’s post hoc test for multiple comparisons, used to compare each treatment group with the control group. Since no significant differences were observed at baseline (*p* > 0.05), a one-way ANOVA was conducted on the post-intervention data to assess the effects of the interventions. Statistical significance was defined as *p* < 0.05 or *p* < 0.01, with “ns” indicating non-significance. All analyses were conducted using GraphPad Prism 9 and SPSS 22.0 software [18].

## 3. Results

### 3.1. Daily Dietary Intake Records

**Figure 6:**
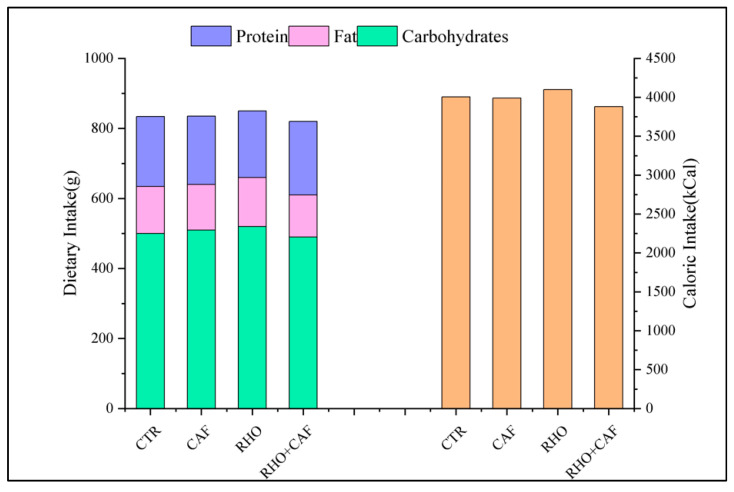
Dietary nutrient intake and composition of subjects.

To ensure consistency and minimize the potential impact of dietary factors, we provided all participants with standardized meal options tailored to meet specific nutritional requirements. Adjusted dietary intake data showed that the CTR group consumed approximately 501 g of carbohydrates, 134 g of fat, and 197 g of protein, with a total caloric intake of about 3999 kcal. Similarly, the CAF group consumed around 509 g of carbohydrates, 131 g of fat, and 193 g of protein, resulting in a total caloric intake of approximately 3982 kcal. The RHO group recorded a dietary intake of approximately 521 g of carbohydrates, 139 g of fat, and 192 g of protein, corresponding to a total caloric intake of about 4098 kcal. Meanwhile, the RHO + CAF group consumed around 494 g of carbohydrates, 117 g of fat, and 208 g of protein, with a total caloric intake of approximately 3860 kcal. These data demonstrate that while minor variations existed between groups, the dietary intake across experimental groups was comparable, ensuring minimal influence of dietary factors on study outcomes. Throughout the study, participants adhered to their habitual dietary patterns, maintaining consistency in their overall intake. Figure 6 illustrates the nutritional intake of subjects.

### 3.2. Outcome of Two-Step-Approach Jump Height Test

**Figure 7:**
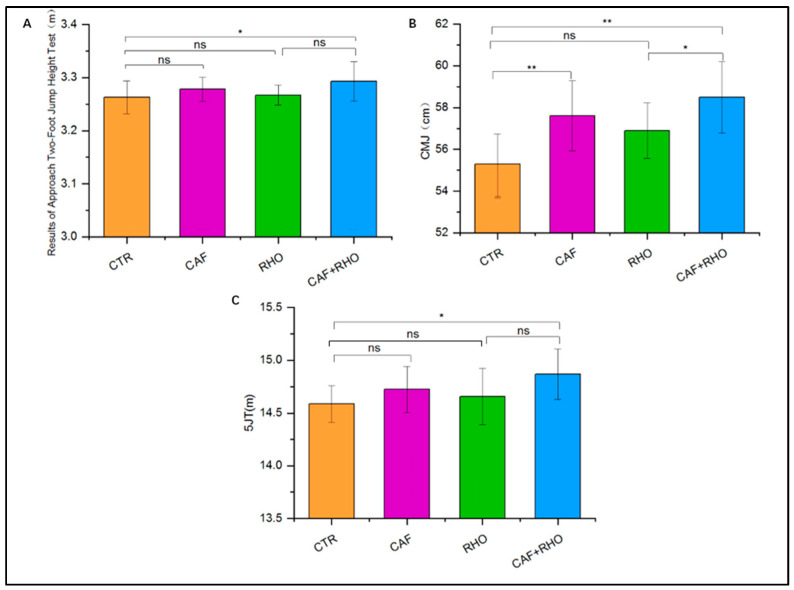
Exercise performance results of subjects (p* < 0.05, p** < 0.01). Results of (**A**) two-step-approach jump height test, (**B**) countermovement jump (CMJ), (**C**) five-jump test (5 JT).

In the two-step-approach jump height test, the control group (CTR) recorded an average jump height of 3.26 ± 0.03 m. No statistically significant differences were observed between the CTR and CAF groups or between the CTR and RHO groups. However, the CAF + RHO group showed notable improvements, achieving the highest jump height (*p* < 0.05). This suggests that combined supplementation may be more effective in enhancing jump height than either supplement alone (Figure 7A).

### 3.3. Outcome of Countermovement Jump (CMJ) Test

In the countermovement jump (CMJ) test, the CTR group recorded an average jump height of 55.3 ± 1.40 cm. Both the CAF and CAF + RHO groups showed significant improvements compared to the CTR group, with the CAF + RHO group achieving the highest CMJ performance (*p* < 0.01 and *p* < 0.05, respectively). These findings suggest that combined supplementation provides a significant advantage in enhancing lower limb explosive power (Figure 7B).

### 3.4. Outcome of Five-Jump Test (5 JT)

The five-jump test (5 JT) provided a comprehensive assessment of explosive lower-limb power. The CTR group recorded an average total distance of 14.58 ± 0.17 m. Neither the CAF nor the RHO group demonstrated a statistically significant improvement compared to the CTR group (*p* > 0.05). However, the CAF + RHO group showed a significant improvement over the CTR group, indicating that CAF + RHO supplementation effectively enhances five-jump performance and contributes to improved lower-limb explosive power (Figure 7C).

### 3.5. Outcome of Continuous 20 Vertical Jumps Test

**Figure 8:**
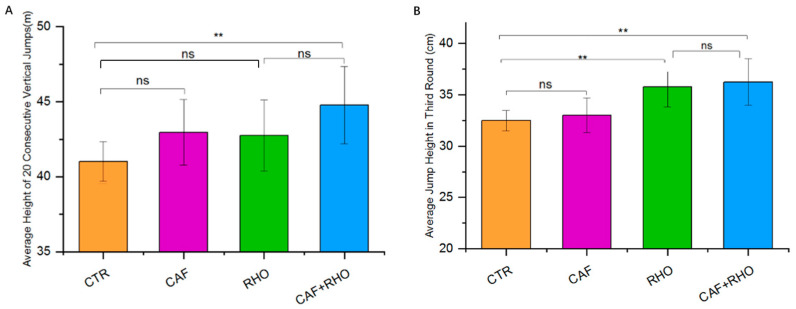
Exercise performance results of subjects (p** < 0.01). (**A**) Average height of 20 consecutive vertical jumps. (**B**) Average jump height in the third round.

In the continuous 20 vertical jumps test, which assesses sustained explosive power, the CTR group recorded an average height of 41.0 ± 1.30 cm. Neither the CAF nor the RHO group demonstrated a significant improvement compared to the CTR group (*p* > 0.05). However, the CAF + RHO group achieved a statistically significant improvement, suggesting that the combined supplementation may be particularly effective in enhancing explosive power during repeated vertical jumps (Figure 8A).

### 3.6. Outcome of Intermittent Jump Recovery Test

In the intermittent jump recovery test, sustained explosive power was assessed across three rounds. In the third round, the CTR group recorded an average height of 32.5 ± 1.0 cm. Both the RHO and CAF + RHO groups showed significant improvements compared to the CTR group, with the CAF + RHO group demonstrating the greatest enhancement over the individual supplementation groups. These findings suggest that combined supplementation may be more effective in sustaining power output across multiple rounds of high-intensity efforts (Figure 8B).

### 3.7. Rate of Perceived Exertion (RPE)

**Figure 9:**
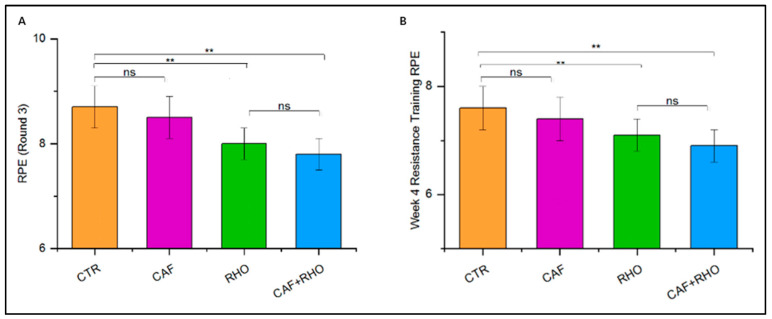
Exercise performance results of subjects (p** < 0.01). (**A**) Rate of perceived exertion (RPE), (**B**) Week 4 resistance training RPE.

As depicted in Figure 9A, no significant difference was observed between the CTR and CAF groups (*p* > 0.05). However, significant differences were found between the CTR group and both the RHO and CAF + RHO groups (*p* < 0.01), indicating that supplementation with RHO alone or in combination with caffeine (CAF + RHO) effectively reduced perceived exertion. Additionally, a significant reduction in perceived exertion was observed in both the RHO and CAF + RHO groups compared to the CTR group (*p* < 0.01) during week 4 of resistance training RPE, further supporting the potential benefits of RHO and CAF + RHO supplementation in mitigating exercise-induced fatigue.

Furthermore, in both the RPE (Round 3) test and the week 4 resistance training RPE test, the CAF + RHO group demonstrated the greatest reduction in perceived exertion. These findings suggest that combined supplementation may be particularly effective in lowering perceived exertion levels, potentially enabling athletes to better manage training loads (Figure 9B).

## 4. Discussion

This study aimed to evaluate the effects of RHO and CAF supplementation on lower limb explosive power and fatigue recovery in volleyball players. The supplementation of RHO and CAF in our study led to significant improvements in lower limb explosive power and fatigue recovery. In this study, we employed a strict control of confounding variables to ensure that the only factor influencing the observed outcomes was supplementation. Training intensity, training experience, diet, and sleep were all carefully controlled, meaning that any observed differences in performance and fatigue recovery could be attributed to the supplementation, supporting our initial hypothesis.

First, the supplementation of RHO showed improvement in anti-fatigue and subjective fatigue perception, as evidenced by a reduction in RPE (Round3) scores, which is consistent with previous studies. Previous studies have shown that RHO may improve muscle endurance by increasing the expression of erythropoietin (EPO) [3,12]. Increased EPO expression is a key factor in enhancing exercise performance, which is consistent with previous research findings [3]. Additionally, the continuous supplementation of RHO significantly increased oxygen consumption in muscle fibers, a key indicator of mitochondrial respiration in skeletal muscle. This improvement suggests that mitochondrial oxidative phosphorylation activity has correspondingly increased, which is particularly beneficial for aerobic exercise, thereby enhancing performance. These findings are in line with previous research results [3,19]. In one study on mice, Hou Y et al. administered RHO orally and after two weeks measured indices including lactate, catalase, and lactate dehydrogenase and observed the ultrastructure of mitochondria. The results showed that the oral RHO solution used in the study was able to inhibit fatigue-induced oxidative stress by increasing superoxide dismutase, catalase, and total antioxidant capacity [20,21]. RHO was able to increase the antioxidant capacity by inhibiting the PTEN-induced kinase 1 (PINK1)–Parkin pathway, increasing the energy produced and inhibiting mitochondrial autophagy, resulting in an anti-fatigue effect [22,23,24].

The level of oxidative stress response substances in skeletal muscle plays an important role in regulating muscle function [25]. RHO supplementation is considered a positive strategy to prevent oxidative stress and improve athletic performance. RHO extract can enhance antioxidant capacity by upregulating the expression of antioxidant enzymes, reducing skeletal muscle damage, and improving the body’s anti-fatigue ability [19,26,27]. Some researchers have suggested that after 30 days of RHO supplementation, lactate dehydrogenase and creatine kinase are reduced after exercise, meaning that muscle damage from strenuous exercise is reduced [28]. Other studies in rats have shown that RHO supplementation 30 min before training promotes mitochondrial recovery of ATP during or after strenuous exercise. Overall, the review suggests that RHO improves antioxidant capacity and reduces muscle damage and fatigue [2]. Similarly, previous studies have shown that individuals taking RHO may experience reduced pain and muscle damage after exercise, thereby improving performance [1,2]. In this study, the RHO group demonstrated significant fatigue tolerance in both the continuous 20 vertical jumps and fatigue recovery tests, with a decrease in subjective fatigue perception (RPE), indicating the potential advantage of RHO in delaying fatigue and promoting recovery.

CAF supplementation also showed a positive impact on explosive power, particularly in the two-step-approach jump height and countermovement jump (CMJ) tests. Numerous studies have confirmed that CAF enhances muscle endurance, speed, and explosive power [29,30,31,32,33]. On one hand, CAF directly stimulates the central nervous system, delaying fatigue and improving alertness and attention [34]. On the other hand, CAF is structurally similar to adenosine, making it an antagonist of adenosine receptors, thereby preventing adenosine from binding to A1 or A2a receptors [35]. This antagonistic effect also promotes the release of neurotransmitters, such as increased dopamine release in the striatum [1,36]. However, some studies have shown that the acute supplementation of 3 mg/kg CAF does not significantly improve certain physical performances, such as CMJ, squat jump, 5–10 m sprints, running speed, or technical movement quality [37,38,39,40]. In this study, although the CAF group demonstrated some improvement in the two-step-approach jump height test and the 5 JT test, the enhancement was not statistically significant compared to the CTR group. This discrepancy may be attributed to the fact that performance in these tests is partially influenced by the athletes’ proficiency in movement techniques, which may have been more developed in the CTR group, thereby masking the effects of CAF supplementation. Additionally, individual genetic variations in caffeine metabolism, such as CYP1A2 genotypes, could also play a role in modulating the efficacy of CAF. For instance, Guest et al. [41] demonstrated that the CYP1A2 rs762551 polymorphism significantly influences the ergogenic effects of CAF, with AA genotype individuals exhibiting notable performance improvements, while CC genotype individuals may experience performance decrements. Therefore, the combined influence of technical proficiency and genetic variability may explain the lack of significant improvement in the CAF group compared to the CTR group.

Most notably, the combined supplementation of RHO and CAF (CAF + RHO group) exhibited the best results in multiple tests, with the highest increase in 20 consecutive vertical jumps, suggesting a possible synergistic effect. One possible explanation is that RHO regulates mitochondrial biogenesis through the AMPK/Sirt1/PGC-1α pathway, with PGC-1α, a downstream component of the AMPK pathway, playing a central role in regulating mitochondrial biogenesis, possibly affecting cellular energy metabolism and muscle endurance [42,43,44,45,46]. Meanwhile, CAF enhances exercise performance by antagonizing adenosine receptors, increasing the excitability of the nervous system, and promoting the release of neurotransmitters [47]. The combination of these dual mechanisms may jointly improve athletic performance at both the physiological and neurological levels.

In the fatigue recovery test, the CAF + RHO group exhibited the lowest decline in power output across three rounds of intermittent jumps. Compared to the CTR group, the CAF + RHO group demonstrated an improvement in jump height, with a statistically significant difference (*p* < 0.01). This suggests that the combined supplementation of caffeine and RHO may contribute to better fatigue resistance and sustained power output during repeated jump efforts. This suggests that the combined supplementation effectively delayed fatigue accumulation and enhanced recovery between high-intensity exercises, as shown by reduced Week 4 Resistance Training RPE ratings in the CAF + RHO group compared to the control group (*p* < 0.01) [48,49]. Furthermore, the RPE ratings during four weeks of resistance training were significantly reduced in the CAF + RHO group, reflecting reduced subjective fatigue. This aligns with previous research findings that RHO supplementation can alleviate fatigue by reducing oxidative stress and enhancing antioxidant capacity [19,26,27]. The addition of CAF may further enhance this effect, enabling athletes to maintain higher performance levels during high-intensity training [50].

It is worth emphasizing that the training protocol in this study adopted a high-intensity lower limb resistance training program based on classic physical training theories, including core movements such as back squats, deadlifts, and box jumps [51]. This training combined improvements in both strength and explosive power, having a significant impact on the comprehensive development of lower limb muscles.

Notably, the CAF + RHO group exhibited a more pronounced reduction in RPE scores compared to the other groups, suggesting that participants in this group adapted more effectively to the training load for reasons that remain unclear. This decrease in RPE may indicate that lower limb resistance training became progressively less challenging for the CAF + RHO group, potentially reflecting an enhanced ability to tolerate higher training intensities.

However, an important limitation of this study is that we did not systematically record and analyze total resistance training volumes, which may introduce minor variability affecting our results. Future research could enhance the accuracy of assessing the effects of RHO and CAF supplementation on training adaptations by systematically tracking training load progression over time. This approach would provide a more comprehensive understanding of how supplementation influences performance improvements and fatigue recovery in relation to training intensity.

However, this study has certain limitations. First, the sample size is relatively small and limited to male volleyball players, which may restrict the generalizability of the results. Future studies should consider expanding the sample to include different genders and athletes from other sports disciplines. Second, we did not measure physiological and biochemical indicators (such as EPO levels, antioxidant enzyme activity, or neurotransmitter concentrations), which limits our ability to explore the specific mechanisms of supplementation effects.

## 5. Conclusions

In summary, this study demonstrated that 30 days of combined supplementation with Rhodiola rosea and caffeine significantly improved lower limb explosive power, reduced training fatigue, and enhanced fatigue recovery in volleyball players. This finding provides a new nutritional strategy for athletes to optimize physical performance during high-intensity training and competition.

## Figures and Tables

**Table 1:** Body characteristics of all participants.

Group (*n* = 12)	Age (Years)	Height (cm)	Weight (kg)	BMI (kg/m^2^)	Training Years (Years)
CTR	20 ± 1	183 ± 5	78 ± 5	23.3 ± 1.2	3.2 ± 0.9
RHO	20 ± 2	183 ± 4	79 ± 5	23.5 ± 1.2	3.3 ± 1.1
CAF	21 ± 1	185 ± 5	78 ± 5	23.2 ± 1.3	3.1 ± 0.8
CAF + RHO	20 ± 1	183 ± 4	79 ± 5	23.4 ± 1.3	3.4 ± 0.9

**Table 2:** Lower limb resistance training program.

Exercise	Sets × Reps	Intensity	Rest Between Sets
Back Squat	6 × 6	75–85% of 1 RM	2 min
Deadlift	5 × 5	70–80% of 1 RM	2 min
Leg Press	4 × 8	60–70% of 1 RM	1 min
Vertical Jump	5 × 20	Bodyweight	1 min
Box Jump	4 × 10	50–60 cm (adjusted)	1 min
Depth Jump	4 × 8	30–40 cm (adjusted)	40 s

Reps: Repetitions; 1 RM: One repetition maximum.

**Table 3:** Baseline values.

Group	Two-Foot Jump Height (m)	CMJ (m)	5 JT (m)	20 J Avg (m)	20 J 3rd Round Avg (m)	20 J 3rd Round RPE	1st Week Resistance Training RPE
CTR	3.23 ± 0.01	0.53 ± 0.04	14.13 ± 0.51	0.41 ± 0.02	0.31 ± 0.01	9.12 ± 0.31	7.62 ± 0.31
CAF	3.24 ± 0.05	0.54 ± 0.05	14.07 ± 0.55	0.39 ± 0.02	0.32 ± 0.01	9.0 ± 0.48	7.5 ± 0.43
RHO	3.24 ± 0.04	0.52 ± 0.06	14.24 ± 0.46	0.42 ± 0.02	0.33 ± 0.02	8.71 ± 0.33	7.42 ± 0.42
CAF + RHO	3.23 ± 0.05	0.52 ± 0.04	14.42 ± 0.62	0.41 ± 0.03	0.31 ± 0.02	9.0 ± 0.48	7.71 ± 0.33

## Data Availability

The original contributions presented in this study are included in the article. Further inquiries can be directed to the corresponding authors.
